# Impact of Medicare Advantage Prescription Drug Plan Star Ratings on Enrollment before and after Implementation of Quality-Related Bonus Payments in 2012

**DOI:** 10.1371/journal.pone.0154357

**Published:** 2016-05-05

**Authors:** Pengxiang Li, Jalpa A. Doshi

**Affiliations:** 1 Division of General Internal Medicine, Perelman School of Medicine, University of Pennsylvania, Philadelphia, PA, United States of America; 2 Leonard Davis Institute of Health Economics, University of Pennsylvania, Philadelphia, PA, United States of America; University of New Mexico Cancer Center, UNITED STATES

## Abstract

**Objective:**

Since 2007, the Centers for Medicare and Medicaid Services have published 5-star quality rating measures to aid consumers in choosing Medicare Advantage Prescription Drug Plans (MAPDs). We examined the impact of these star ratings on Medicare Advantage Prescription Drug (MAPD) enrollment before and after 2012, when star ratings became tied to bonus payments for MAPDs that could be used to improve plan benefits and/or reduce premiums in the subsequent year.

**Methods:**

A longitudinal design and multivariable hybrid models were used to assess whether star ratings had a direct impact on concurrent year MAPD contract enrollment (by influencing beneficiary choice) and/or an indirect impact on subsequent year MAPD contract enrollment (because ratings were linked to bonus payments). The main analysis was based on contract-year level data from 2009–2015. We compared effects of star ratings in the pre-bonus payment period (2009–2011) and post-bonus payment period (2012–2015). Extensive sensitivity analyses varied the analytic techniques, unit of analysis, and sample inclusion criteria. Similar analyses were conducted separately using stand-alone PDP contract-year data; since PDPs were not eligible for bonus payments, they served as an external comparison group.

**Result:**

The main analysis included 3,866 MAPD contract-years. A change of star rating had no statistically significant effect on concurrent year enrollment in any of the pre-, post-, or pre-post combined periods. On the other hand, star rating increase was associated with a statistically significant increase in the subsequent year enrollment (a 1-star increase associated with +11,337 enrollees, p<0.001) in the post-bonus payment period but had a very small and statistically non-significant effect on subsequent year enrollment in the pre-bonus payment period. Further, the difference in effects on subsequent year enrollment was statistically significant between the pre- and post-periods (p = 0.011). Sensitivity analyses indicated that the findings were robust. No statistically significant effect of star ratings was found on concurrent or subsequent year enrollment in the pre- or post-period in the external comparison group of stand-alone PDP contracts.

**Conclusion:**

Star ratings had no direct impact on concurrent year MAPD enrollment before or after the introduction of bonus payments tied to star ratings. However, after the introduction of these bonus payments, MAPD star ratings had a significant indirect impact of increasing subsequent year enrollment, likely via the reinvestment of bonuses to provide lower premiums and/or additional member benefits in the following year.

## Introduction

In the United States, Medicare is a national social insurance program for the elderly and disabled that has been administered by the federal government since 1966. It provides health insurance for more than 55 million Americans [1]. In 2014, approximately 30% of Medicare beneficiaries (16 million) were enrolled in Medicare Advantage plans that allow private insurers to provide Medicare-covered benefits to Medicare enrollees, with regulation by the federal government [2]. Most Medicare Advantage plans include Part D prescription drug benefits and are known as Medicare Advantage Prescription Drug plans (MAPDs) [3].

Since 2007, the Centers for Medicare and Medicaid Services (CMS) have publicly posted quality ratings of Medicare plans (including MAPD plans and stand-alone Part D prescription drug plans [PDPs]) on a 1- to 5-star scale, with 5 stars representing excellent performance and 1 star representing poor performance. Ratings are based on the plan’s performance data from a prior 18-month period (e.g., January 2012 to June 2013 plan performance determined the 2014 star rating). For both Medicare Advantage plans and stand-alone PDPs, sponsoring organizations (e.g., Aetna, Humana) offer contracts to CMS that include one or more plans, of varying types (e.g., health maintenance organization [HMO], preferred provider organization [PPO]), for specific geographic areas. CMS calculates star ratings at the contract level [4,5]. Contracts that are too new to be measured or do not have enough data available are not rated [5]. CMS releases star ratings for the current plan enrollment year in the second half of the previous year so that they are available during Medicare’s open enrollment period to inform beneficiaries’ plan choices. For example, CMS released 2014 star ratings in the second half of 2013 so that consumers could use this information during the open enrollment period to inform their enrollment in a plan beginning January 2014. The star rating for MAPD plans includes measures of health services quality (Part C rating: e.g., Staying Healthy [screenings, tests, and vaccines], Managing Chronic Conditions) and drug services quality (Part D rating: e.g., member experience with the plan’s drug services).

The Star Ratings program is intended to provide quality information that will enable Medicare beneficiaries to make more informed enrollment decisions. However, initial reports on the influence of star ratings on plan enrollment have been mixed. Surveys have suggested that star ratings play little role in seniors’ plan choices [6,7]. At the same time, quantitative empirical studies have indicated that higher star ratings were in fact associated with higher MAPD enrollment [4,8,9]. However, these studies all used cross-sectional study designs and thus could not establish a causal link between ratings and increased enrollment. They also did not rule out potential confounders that could have influenced enrollment decisions, including plan reputation or the size or quality of provider networks [10–12]. For example, a plan with a high star rating may also have greater name recognition, which could result in a competitive advantage and higher enrollment that might not be due to the high rating alone. Hence, prior studies are limited in their assessment of the direct impact of star ratings on concurrent year enrollment.

**Fig 1:**
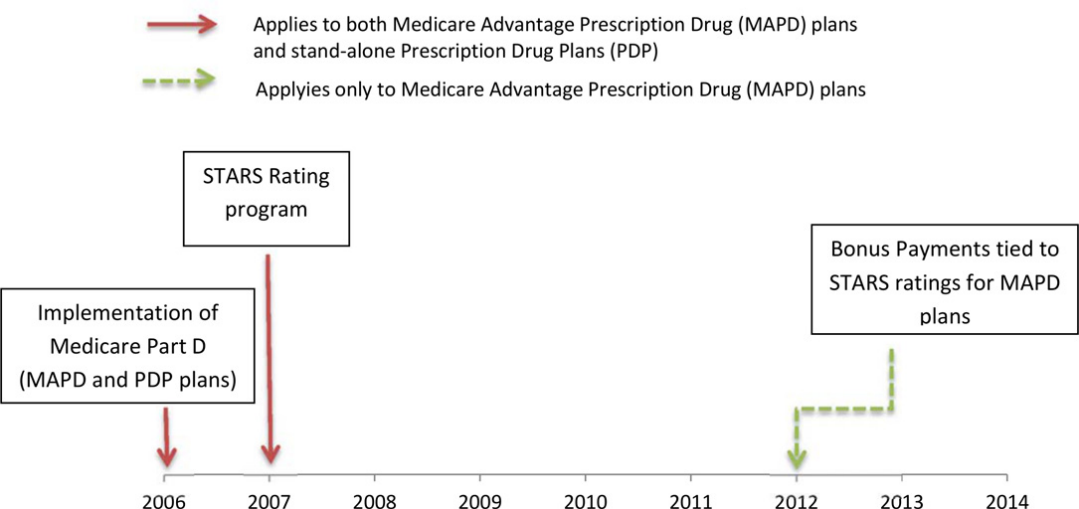
Timeline of Medicare Plan Quality Rating and Pay for Performance System.

**Fig 2:**
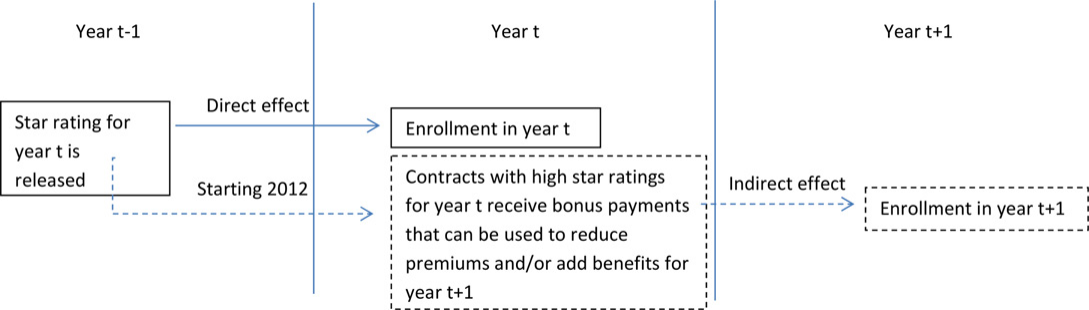
Hypothesized Direct and Indirect Relationships Between MAPD Contract Star Ratings and Enrollment.

Perhaps most importantly, prior studies have not examined the impact of more recent changes to the Star Ratings program that may indirectly enhance the impact of star ratings on enrollment in the subsequent year (Figs 1 and 2). Starting in 2012, provisions of the Affordable Care Act and a CMS demonstration program began to tie plan ratings to bonus payments for MAPD plans. Plans with higher ratings (3 or more stars through the CMS demonstration project and 4 or more stars through the Affordable Care Act provisions) became eligible to receive quality bonus payments (QBPs), which were then required to be invested in additional benefits for enrollees (e.g., paying for transportation to medical appointments) and/or to reduce premiums in the subsequent year (i.e., bonus payments tied to 2014 star ratings would affect plan benefits and/or premiums in 2015).

In summary, additional research is needed not only to tease out the direct impact of star ratings on concurrent year plan enrollment but also their indirect impact (via bonus payments) on subsequent year plan enrollment (Fig 2). Our study sought to address these gaps in the literature by examining the impact of star rating changes on enrollment changes using a longitudinal study design. This is the first longitudinal study to examine the impact of CMS star ratings on enrollment and to assess how the impact differed before and after implementation of these bonus payments.

## Methods

### Study Design and Sample

This was a longitudinal study to examine the impact of star rating changes within MAPD contracts on changes in MAPD contract enrollment, before and after bonus payments were introduced in 2012. The pre-period was defined as 2009 to 2011 and the post-period was defined as 2012 to 2015. CMS calculates star ratings at the contract level, and these ratings apply to all plans that fall within that contract [4]. Thus, we examined star rating and enrollment changes at the contract level in our main analysis, with contract-years as the unit of analysis.

In the main analysis, the MAPD group included health maintenance organization (HMO), point-of-service (POS), local preferred provider organization (PPO), private fee-for-service (PFFS), and regional PPO contract types. Since so-called Medicare Cost contracts (managed care plans paid on the basis of the reasonable costs of delivering Medicare-covered services) were not assigned star ratings prior to 2012, they were excluded from the main analysis but were included in sensitivity analyses.

### Data Source

The primary sources of data were 2009 to 2015 CMS Part C and Part D Performance Data [13] and Medicare Advantage/Part D Contract and Enrollment Data [14]. Star ratings for MAPD and PDP contracts were retrieved from CMS Part C and D Performance Data [13]. Contract and plan enrollment and characteristics data were retrieved from Medicare Advantage/Part D Contract and Enrollment Data [14]. Enrollment data were based on January enrollment, given that this is the first month after the open enrollment period.

### Outcomes

As explained in the introduction section, star ratings for year t were released in year t-1 so that they would be available to inform consumer decision making during Medicare’s open enrollment period, when consumers were choosing their plan for year t. (For example, 2014 MAPD star ratings were available in 2013, for Medicare enrollees to choose their 2014 plan.) However, bonus payments tied to year t’s star ratings were received by plans during year t and could be used to improve plan benefits and/or lower premiums for the subsequent year t+1. Hence, we used two outcome variables in our study. The first was enrollment (total number of enrollees in a contract or plan) in the concurrent year t, to test the direct impact of star ratings for year t in informing beneficiary enrollment choices. The second was enrollment (total number of enrollees in a contract or plan) in the subsequent year t+1, to test the indirect impact of the star ratings for year t via the bonus payments tied to the star ratings that were introduced in 2012 (Fig 2).

### Star Ratings

Overall ratings (representing a combination of Part C and Part D ratings) for MAPD plans were available from 2011 to 2015. In 2009 and 2010, MAPDs only had separate ratings for Part C and Part D. Following the procedures used by the Medicare Payment and Advisory Commission (MedPAC) [15], we simulated an overall rating for MAPDs in 2009 and 2010 using the same methodology that was used to create the 2011 overall rating [16]. As has been done in prior studies [4,8], we coded star ratings as a continuous variable to facilitate parsimonious model specifications and ease of interpretation of the results (e.g., effect of a 1-star increase in rating on MAPD enrollment).

### Control variables

Control variables in all multivariable models at the contract-year level analysis included contract types (health maintenance organization (HMO), point-of-service (POS), local provider organization (PPO), private fee-for-service (PFFS), regional PPO, or Medicare Cost [when included]), contract maturity (how many years the contract has been in existence, defined as the time elapsed between the year the contract became effective and the study year), and the lagged (t-1 year) variables of total number of plans in the contract, proportion of plans in the contract that offered Part D coverage, proportion of plans in the contract that were Special Needs Plans ([SNPs] i.e., those that limit membership to people with specific diseases or characteristics, and tailor their benefits, provider choices, and drug formularies to best meet the specific needs of the groups they serve), and the proportion of plans in the contract that were employer group health plans in the prior (t-1) year. For plan-year level analyses conducted as part of sensitivity analyses, the control variables were plan types, contract maturity, year dummy variable, and premium amount. Premium amount included Part C and D premiums [17]. All models also included year dummy variables to capture time trends.

### Statistical analyses

Multivariable panel data hybrid models [18] were used for the main analyses. These models decompose each time-varying covariate (i.e., star rating, number of plans in the contract, percentage of plans offering Part D, percentage of plans that were SNPs, and percentage of plans that were employer group health plans) into two parts: the between part (mean of the variable over time) and the within part (the difference between the value of the variable in the period of interest and the mean of the variable over time). In addition, time-invariant variables can also be included in the model. Hybrid models allowed us to test how differences in star ratings *between* contracts were associated with enrollment differences between them (e.g., the difference in enrollment between two contracts with a 1-star difference in their star rating) and how changes in star ratings *within* the same contract across years was associated with changes in its enrollment (e.g., the changes in enrollment after a 1-star increase in a contract’s star rating). It should be noted that the “*between effect”* estimates from these models represent the cross-sectional association between star ratings and enrollment, which is prone to bias due to unmeasured differences across contracts. On the other hand, the “*within effect”* estimates from the same models can yield unbiased effects of star rating changes on enrollment by ruling out the impact of unmeasured time-invariant confounders (e.g., plan’s name recognition) and thus represent our main results of interest in this longitudinal study [18,19]. Two sets of the same model specification were estimated for our two different outcomes (i.e., enrollment in concurrent year t and subsequent year t+1). Models were estimated separately on data from the pre-bonus payment period (2009–2011) and the post-bonus payment period (2012–2015) to identify if there was a differential impact of star ratings on subsequent year enrollment due to reinvestment of bonuses to improve plan benefits and/or reduce premiums in the post-period. Models were also estimated on the combined pre- and post-bonus payment period years data, wherein we also included a variable to indicate the post-bonus payment period (2012 or later) and an interaction term between the post-period and star rating variables to test for statistical differences in the impact of star ratings on enrollment between the pre- and post-bonus payment periods.

We conducted extensive sensitivity analyses by varying the analytic technique, unit of analysis, and sample selection criteria. First, multivariable contract-level fixed effects models [19] instead of hybrid models were used to test the robustness of the findings. Second, sensitivity analyses were performed using alternative inclusion and exclusion criteria for the type of contracts included in the contract-year level study sample, namely (i) including Medicare Cost contracts; (ii) excluding PFFS contracts; and (iii) excluding contracts with a high percentage of SNPs or employer group health plans. Third, we conducted a sensitivity analysis using the plan-year level as the unit of analysis. SNPs and employer group health plans were excluded from plan-level analyses given that they are characterized by restricted choice and are different from general MAPD plans. Two sets of plan-year level models were run; one without plan premium variables and another set including plan premium variables. In post-hoc analyses, we also examined how star rating changes impacted current year premiums and subsequent year premiums at the plan level.

To further understand whether differences in the impact of star ratings on MAPD enrollment before and after 2012 were likely due to the bonus payments, we repeated our analysis in an external comparison group of stand-alone PDP contracts, which also received star ratings throughout the study period (2009–2015) but were not eligible for bonus payments (see Fig 1). For stand-alone PDP contracts, only the Part D star rating is available since PDP plans only provide drug benefits [3], whereas for MAPD contracts the star rating combines Part C and D ratings since MAPD plans provide both medical and drug benefits. Given that the star ratings for the two types of contracts differed in the services for which quality is captured and also the factors influencing enrollment choices for a drug-only plan versus a medical-plus-drug plan may vary, we did not pool the stand-alone PDP and MAPD contract data to conduct a formal statistical test of differences in differences. Instead, multivariable hybrid models were estimated separately in the stand-alone PDP contract sample with models estimated separately on data from the pre-bonus payment period (2009–2011), the post-bonus payment period (2012–2015), and the pre-post combined period (2009–2015).

All the models were estimated using STATA Version 14.0 and adjusted for repeated measures over time.

### Ethics statement

The study was reviewed and approved by the Institutional Review Board of the University of Pennsylvania. Since no data were collected directly from human subjects, informed consent was not required.

## Results

From 2009 to 2015, there were 3,866 contract-years with mean enrollment of 22,883 beneficiaries (SE = 921). Unrated contracts had the lowest enrollment, with a mean of 4,259 beneficiaries (SE = 639). In general, higher ratings were associated with higher enrollment, a greater number of plans in the contract, and longer contract maturity. For example, contracts with ratings of 2.5 stars or fewer had mean enrollment of 16,222 beneficiaries, a mean of 7 plans, and had been in business for an average of 6 years. On the other hand, contracts with ratings of 5 stars had mean enrollment of 126,345 beneficiaries, a mean of 12 plans, and had been in business for an average of 20+ years. In addition, contracts with higher ratings tended to have a lower percentage of SNPs and a higher percentage of employer group health plans. About 67% of overall contracts were HMO or POS, 26% of overall contracts were local PPO, and 100% of 5-star contracts were HMO or POS (Table 1).

**Table 1:** Sample Characteristics by CMS Star Ratings.

		CMS Star Ratings				
	Total	Unrated	2.5 or fewer	3 to 3.5	4 to 4.5	5
Number of contract-years	3,866	1,150	305	1,652	717	42
Contract enrollment, mean (SE)	22,883 (921)	4,259 (639)	16,222 (2,236)	27,303 (1,151)	39,343 (2,870)	126,345 (39,841)
Total number of plans, mean (SE)	6.60 (0.14)	4.62 (0.01)	7.36 (0.01)	6.86 (0.00)	8.55 (0.01)	12.19 (0.02)
Proportion of plans in contract offering Part D, mean (SE)	0.84 (0.00)	0.86 (0.01)	0.88 (0.01)	0.84 (0.00)	0.79 (0.01)	0.73 (0.02)
Proportion of plans in contract that were SNPs, mean (SE)	0.25 (0.01)	0.31 (0.01)	0.37 (0.02)	0.24 (0.01)	0.14 (0.01)	0.04 (0.02)
Proportion of plans in contract that were EGHPs, mean (SE)	0.25 (0.00)	0.23 (0.01)	0.18 (0.02)	0.25 (0.01)	0.31 (0.01)	0.38 (0.04)
Contract maturity^a^, mean (SE)	7.84 (0.11)	2.66 (0.08)	6.28 (0.20)	9.39 (0.16)	12.52 (0.28)	20.17 (1.55)
Contract type, No (%)						
HMO/HMO-POS	2,579 (66.7)	709 (61.7)	227 (74.4)	1,078 (65.3)	523 (72.9)	42 (100.0)
Local PPO	1,008 (26.1)	318 (27.7)	50 (16.4)	458 (27.7)	182 (25.4)	0 (0.0)
PFFS	193 (5.0)	113 (9.8)	15 (4.9)	56 (3.4)	9 (1.3)	0 (0.0)
Regional PPO	86 (2.2)	10 (0.9)	13 (4.3)	60 (3.6)	3 (0.4)	0 (0.0)

CMS, Centers for Medicare and Medicaid Services; EGHP, employer group health plan; HMO, health maintenance organization; PFFS, private fee-for-service; POS, point of service; PPO, preferred provider organization; SE, standard error of mean; SNP, Special Needs Plan.

^a^MAPD plans first became available in 2006 when the Medicare Part D prescription drug program was implemented and Medicare Advantage plans started offering prescription drug benefits. However, the contract maturity reflects the maturity of the Medicare Advantage plan, which existed before 2006.

**Fig 3:**
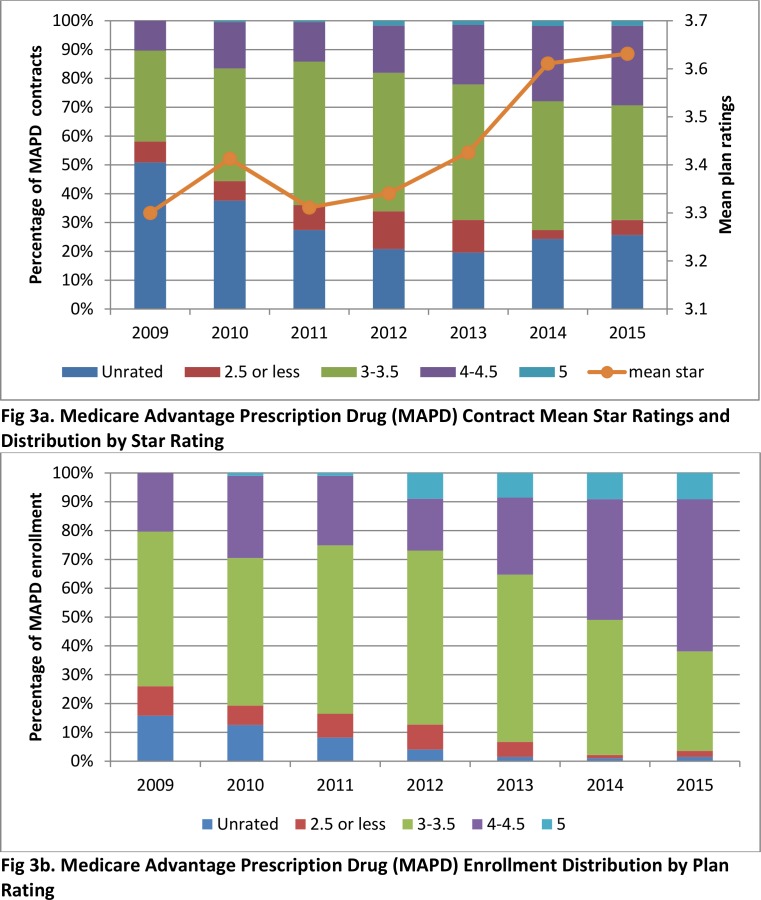
Medicare Advantage Contracts Star Ratings and Enrollment: 2009 to 2015.

Fig 3 shows trends in star ratings and enrollment for Medicare Advantage contracts from 2009 to 2015. Since 2012, a higher proportion of contracts have had higher star ratings and the mean star ratings have increased over time (difference in mean ratings between pre- and post-bonus payment period was 0.16 [p<0.001]). In addition, an increasing proportion of MAPD enrollees were in contracts with higher ratings. Trends were more dramatic in terms of enrollment than number of contracts. For example, the proportion of MAPD contracts with 4 stars or more increased from 18% in 2012 to 30% in 2015, and the proportion of MAPD enrollees in contracts with 4 stars or more increased from 27% in 2012 to 62% in 2015. On the other hand, there was not much change in enrollment in relation to ratings before 2012.

Table 2 presents the results of our multivariable panel data hybrid models, which separate out the effects for between contract differences in star ratings (i.e., between effects) from the effects of within contract changes in star ratings (i.e., within effects) on concurrent and subsequent year enrollment. In general, the between effects were statistically significant across models for both enrollment outcomes and findings indicated that a contract with a higher rating had larger enrollment compared to another contract with a lower rating. For example, based on models including data from the pre-post combined period (2009 to 2015), a 1-star difference in rating between contracts was associated with a difference of 8,089 enrollees (p = 0.042) in concurrent year enrollment and a difference of 9,750 enrollees (p = 0.033) in subsequent year enrollment. These between contract effects, which examined the cross-sectional differences in star ratings between contracts, existed in both the pre- (2009 to 2011) and post-bonus payment periods (2012 to 2015).

**Table 2:** Association of Star Rating and MAPD Contract Enrollment Before and After 2012 (Panel Data Hybrid Model)^a^.

	Enrollment in concurrent year (t)		Enrollment in subsequent year (t+1)	
	Estimate (95% CI)	p-value	Estimate (95% CI)	p-value
Pre-bonus payment period (t = 2009 to 2011)				
Between effect: Star rating difference between contracts	7,858.8 (-335.2, 16,052.7)	0.060	9,280.1 (364.2, 18,195.9)	0.041
**Within effect: Star rating change within a contract**	**148.4 (-4,734.0, 5,030.9)**	**0.952**	**692.5 (-2,109.4, 3,494.4)**	**0.628**
Post-bonus payment period (t = 2012 to 2015)				
Between effect: Star rating difference between contracts	8,322.2 (153.7, 16,490.7)	0.046	10,849.9 (967.9, 20,731.8)	0.031
**Within effect: Star rating change within a contract**	**3,558.5 (-55.9, 7,172.9)**	**0.054**	**11,337.1 (6,025.4, 16,648.8)**	**<0.001**
Pre-post combined period (t = 2009 to 2015)				
Between effect: Star rating difference between contracts	8,088.5 (287.1, 15,889.8)	0.042	9,749.6 (798.8, 18,700.4)	0.033
Within effect * Post^a^	**1,594.4 (-4962.6, 8,151.4)**	**0.634**	**9,136.9 (2,106.7, 16,167.1)**	**0.011**

CI, confidence interval; MAPD, Medicare Advantage Prescription Drug plan.

^a^Post = 1 if year was from 2012 to 2015 and Post = 0 if year was from 2009 to 2011. Estimates were based on random effects models adjusted for contract-level repeated measures and controlling for contract types (health maintenance organization [HMO], point-of-service [POS], local provider organization [PPO], private fee-for-service [PFFS], or regional PPO), contract maturity (how many years the contract has been in existence, defined as the time elapsed between the year the contract became effective and the study year), and the lagged (t-1 year) variables of total number of plans in the contract, proportion of plans in the contract that offered Part D coverage, proportion of plans in the contract that were Special Needs Plans (i.e., those that limit membership to people with specific diseases or characteristics, and tailor their benefits, provider choices, and drug formularies to best meet the specific needs of the groups they serve), and the proportion of plans in the contract that were employer group health plans in the prior (t-1) year, and year dummy variables. “Within effect * Post” is the interaction term between “Within effect” and “Post.”

On the other hand, within contract effects, which examined the effect of longitudinal changes in star ratings within contracts, were substantially different across the two enrollment outcomes and pre-post time periods. Firstly, a change of star rating had no statistically significant effect on concurrent year enrollment in any of the pre-, post-, or pre-post combined periods. Secondly, star rating increase was associated with a statistically significant increase in the subsequent year enrollment (a 1-star increase associated with +11,337 enrollees, p<0.001) in the post-bonus payment period (2012 to 2015) but had a very small and statistically non-significant effect on subsequent year enrollment in the pre-bonus payment period (2009 to 2012). Further, the interaction term testing the difference in within effects on the subsequent year enrollment between the post- and pre-bonus payment period was statistically significant (p = 0.011).

Sensitivity analyses based on contract-year level panel data fixed effects models also showed results consistent with the main findings (Table A in S1 File). Sensitivity analyses based on contract-year level panel data using alternative sample inclusion criteria also showed findings consistent with the main findings (Tables B-D in S1 File). Sensitivity analyses conducted at the plan-year level showed findings consistent with the main results. After adjusting for premium, a star rating change did not have a statistically significant impact on current year (t) and subsequent year enrollment (t+1), even during the post-period when bonus payments were in effect (Table E in S1 File). Premium amount had a negative impact on enrollment (e.g., every $1 increase in Part C premium was associated with a decrease of 38 to 39 enrollees per plan and every $1 increase in Part D premium was associated with a drop of 46 to 55 enrollees per plan; p<0.001, data not shown). In post-hoc analyses, we did see statistically significant drops in premiums (Part C premium plus Part D premium) in the subsequent year (a 1-star increase in year t was associated with a $4.80 drop in premium in year t+1; p<0.001) during the post-bonus payment period. An increase in star rating was not associated with a subsequent decrease in premium during the pre-bonus payment period (Table F in S1 File).

Finally, similar analyses conducted in our external comparison group of stand-alone Part D plans, wherein bonus payments were not tied to their star ratings, provided further support for our findings on the indirect impact of MAPD star ratings (via bonus payments) on subsequent year enrollment. Unlike MAPD plans, there were no trends of increased enrollment for stand-alone PDPs with high ratings (4 stars or more) after 2012 (Fig A in S1 File). Furthermore, multivariate panel data hybrid models estimated in the sample of stand-alone PDPs did not show statistically significant effects on concurrent year enrollment or subsequent year enrollment during either the pre- or post- bonus payment periods (Table G in S1 File).

## Discussion

CMS’s Medicare Star Rating program was designed to offer guidance to beneficiaries regarding the quality of available Medicare Advantage (MAPD) and stand-alone prescription drug plans (PDPs). In theory, if everything else is equal, we would expect enrollees to prefer higher quality (higher star rating) plans over lower rated plans, which should result in a positive correlation between star ratings and contract enrollments. Although beneficiary surveys and focus groups have called the influence of star ratings on beneficiary enrollment choices into question [6,7], other empirical studies employing cross-sectional designs have found a positive correlation between star rating and contract enrollment [4,8,9]. However, the apparent cross-sectional relationship between star ratings and enrollment may be due to spurious factors such as plan reputation (i.e., highly rated plans also tend to have been in business for a longer period of time and have greater name recognition). Consistent with that prior literature, our descriptive analyses and the “between effects” from our multivariable hybrid models, which captured the cross-sectional differences in star ratings between contracts, also found a positive correlation between star rating and enrollment. However, our study was the first to use longitudinal analyses and to identify “within effects” from multivariable hybrid models to help untangle what factors are driving this correlation between star ratings and enrollment. In fact, our findings suggest that star ratings had no direct impact on concurrent year enrollment before or after the introduction of the bonus payment system in 2012. Rather, MAPD star ratings had a significant indirect impact of increasing subsequent year enrollment, but only after implementation of the bonus payment system. Extensive sensitivity analyses and analyses in an external control group of stand-alone PDPs that were not eligible for bonus payments confirmed the robustness of our findings.

Thus, our findings that the direct impact of star ratings was limited both before and after 2012 is consistent with beneficiary surveys suggesting little change in the degree to which beneficiaries were using star rating information across our study period. A 2011 survey conducted by Harris Interactive on behalf of Kaiser Permanente [6] found that among 483 Medicare-eligible seniors, only one third of them had heard of Medicare plan ratings and 97% of them did not know the rating of their own plan. In 2013, Jacobson et al. [7] conducted focus groups to explore factors affecting plan choice and also found that star ratings did not play a substantial role in seniors’ plan choices. Many Medicare beneficiaries were unaware of the ratings and others questioned whether the ratings captured the coverage issues of greatest concern to them. In addition, information overload and resistance to changing plans were cited as significant issues for beneficiaries, which is perhaps not surprising given the tremendous complexity of the Medicare landscape. This suggests that seniors may benefit from a more patient-centered, interactive rating system that allows them to place greater weight on the issues of greatest concern to them (e.g., coverage of a specific medication, out-of-pocket cost requirements) [7].

Although star ratings do not seem to be serving their intended function in regard to directly aiding plan selection, the associated financial incentives have increased their utility. It is notable that an increase in a contract’s star rating led to a significant increase in enrollment in the subsequent year. These lagged effects may be due to features of the rating program’s incentive system. First, bonus payments must be directed toward enhancing member benefits and/or used to reduce premiums [11,20]. Prior research has found that plan premiums and benefits are the most important factors in Medicare beneficiaries’ decision making regarding plan selection [7,10,12]. Thus, plans with higher star ratings may be able to attract more enrollees in the subsequent year because by that time, bonus payments have been reinvested to offer a more attractive set of benefits and/or lower premiums [11,20]. It was estimated that MAPD plans received $3.1 billion in bonuses in 2012, with about one third of bonus money given to 74 contracts with ratings of 4 stars or higher [11]. In general, plans with higher ratings received larger bonuses [3,11,20–22]. Our post-hoc analysis confirmed that an increase in star rating was indeed associated with a lower premium in the subsequent year during the post-bonus payment period (Table F in S1 File). In addition, in models adjusting for premiums, lower premium amount was associated with increased enrollment and the impact of star ratings on subsequent year enrollment during the post-period became smaller and not statistically significant. This suggests that the premium amount mediates the relationship between star rating for year t (star rating t) and subsequent year enrollment (enrollment t+1) and that the lowered premiums associated with star rating bonus payments play an important role in attracting enrollees to plans with high star ratings during the post-bonus payment period. Second, 5-star MAPDs and PDPs have a competitive advantage in the form of expanded marketing privileges; they are permitted to market their plans to beneficiaries throughout the calendar year whereas other plans can advertise only during the annual open enrollment period. Furthermore, 5-star plans are highlighted with a special icon on the Medicare.gov website in order to encourage enrollment in plans that have met CMS’s definitions of quality [11,23]. At the same time, the website also discourages enrollment in plans with lower ratings (i.e., fewer than 3 stars) by assigning them a warning symbol. Finally, individuals who are enrolled in plans with low ratings are notified and given the opportunity to change to a higher-rated plan. As of 2014, plans failing to achieve a minimum rating of 3 stars for 3 consecutive years are not permitted to enroll beneficiaries through the Medicare website and may be dropped from Medicare [3]. Although these factors could have been driving the association between star ratings and MAPD enrollment, both the extended marketing period and website icons are in effect for stand-alone PDP plans as well–yet we did not observe a corresponding relationship between improved star ratings and increased enrollment among stand-alone PDP contracts. This suggests that MAPD star ratings had a significant indirect impact of increasing subsequent year enrollment, likely via the reinvestment of bonuses to provide lower premiums and/or additional member benefits in the following year.

### Limitations

Our study has several limitations. First, our main analysis using contract-year level data did not include some variables that are relevant to enrollees’ decision making, including plan reputation, premium amounts, specific benefits, and volume or effectiveness of marketing efforts [7,10,12], because the information was not available. Any effects of these variables would be expected to be present across stand-alone PDPs as well, however, and we found no significant relationship between star rating changes and enrollment in those plans. Furthermore, we conducted extensive sensitivity analyses to reduce the influence of unmeasured confounders. We used fixed effects and hybrid models to control for unmeasured time-invariant confounders such as plan reputation, which would not be expected to vary substantially from year to year. We also included premium amounts in sensitivity analyses, which showed consistent findings (i.e., that MAPD star ratings did not have a direct impact on enrollment). Second, we used enrollment numbers instead of market share as our outcome variable. One concern is that the enrollment numbers would automatically increase if the market size increases. However, our use of hybrid/fixed effects models does control for market size because we examine how changes in star ratings are associated with changes in enrollment numbers. As a result of this analytic approach, the influence of market size will be largely differentiated out. Even in a market with a substantial change in market size, plans with no changes in star ratings will serve as contemporaneous controls to control for market size changes not associated with star rating changes. Finally, some contracts merged or went out of business during our study period, which could have biased our results if the merging was systematically associated with plan ratings. Once again, however, this would be expected to affect stand-alone PDPs as well, since most large plan sponsors offer both MAPD and PDP options.

### Conclusions

Star ratings had no direct impact on concurrent year MAPD enrollment before or after the introduction of bonus payments tied to star ratings. However, after the introduction of these bonus payments, MAPD star ratings had a significant indirect impact of increasing subsequent year enrollment, likely via the reinvestment of bonuses to provide lower premiums and/or additional member benefits in the following year.

## Supporting Information

S1 File Supporting file including Table A-G and Fig A. Table A: Sensitivity Analysis on Analytical Technique: Association of Star Rating and MAPD Contract Enrollment Before and After 2012 (Panel Data Fixed Effect Model). Table B: Sensitivity Analysis on Sample Selection: Association of Star Rating and MAPD Contract Enrollment Before and After 2012 (Panel Data Hybrid Model; Including Medicare Cost Contracts in the Sample). Table C: Sensitivity Analysis on Sample Selection: Association of Star Rating and MAPD Contract Enrollment Before and After 2012 (Panel Data Hybrid Model; Excluding PFFS From the Sample). Table D: Sensitivity Analysis on Sample Selection: Association of Star Rating and MAPD Contract Enrollment Before and After 2012 (Panel Data Hybrid Model; Excluding Contracts with High Percentage of Special Needs Plans or Employer Group Health Plans From the Sample). Table E: Sensitivity Analysis on Unit of Analysis: Association of Star Rating and MAPD Plan Enrollment Before and After 2012 (Panel Data Hybrid Model; Plan-Year Level Analysis). Table F: Post-hoc Analysis: Association of Star Rating and MAPD Plan Premium Before and After 2012 (Panel Data Hybrid Model; Plan-Year Level Analysis). Fig A: Stand-Alone Prescription Drug Plan (PDP) Contracts Star Ratings and Enrollment: 2009 to 2015. Table G: Analysis on External Comparison Group: Association of Star Rating and Stand-Alone PDP Contract Enrollment Before and After 2012 (Panel Data Hybrid Model).
(DOCX)

S2 File The analytical file for the main sample. (DTA)
