# Supplementary material for: Impact of Medicare Advantage Prescription Drug Plan Star Ratings on Enrollment before and after Implementation of Quality-Related Bonus Payments in 2012
Source: PLoS One. 2016 May 5;11(5):e0154357. doi: 10.1371/journal.pone.0154357 (PMC4858248; doi:10.1371/journal.pone.0154357)
Supplement: S1 File — Supporting file including Table A-G and Fig A. Table A: Sensitivity Analysis on Analytical Technique: Association of Star Rating and MAPD Contract Enrollment Before and After 2012 (Panel Data Fixed Effect Model). Table B: Sensitivity Analysis on Sample Selection: Association of Star Rating and MAPD Contract Enrollment Before and After 2012 (Panel Data Hybrid Model; Including Medicare Cost Contracts in the Sample). Table C: Sensitivity Analysis on Sample Selection: Association of Star Rating and MAPD Contract Enrollment Before and After 2012 (Panel Data Hybrid Model; Excluding PFFS From the Sample). Table D: Sensitivity Analysis on Sample Selection: Association of Star Rating and MAPD Contract Enrollment Before and After 2012 (Panel Data Hybrid Model; Excluding Contracts with High Percentage of Special Needs Plans or Employer Group Health Plans From the Sample). Table E: Sensitivity Analysis on Unit of Analysis: Association of Star Rating and MAPD Plan Enrollment Before and After 2012 (Panel Data Hybrid Model; Plan-Year Level Analysis). Table F: Post-hoc Analysis: Association of Star Rating and MAPD Plan Premium Before and After 2012 (Panel Data Hybrid Model; Plan-Year Level Analysis). Fig A: Stand-Alone Prescription Drug Plan (PDP) Contracts Star Ratings and Enrollment: 2009 to 2015. Table G: Analysis on External Comparison Group: Association of Star Rating and Stand-Alone PDP Contract Enrollment Before and After 2012 (Panel Data Hybrid Model). (DOCX) [file pone.0154357.s001.docx]

Table A. Sensitivity Analysis on Analytical Technique: Association of Star Rating and MAPD Contract Enrollment Before and After 2012 (**Panel Data Fixed Effect Model**)

|  | Enrollment in concurrent year (t) | | Enrollment in subsequent year (t+1) | |
| --- | --- | --- | --- | --- |
|  | Estimate (95% CI) | P-value | Estimate (95% CI) | P-value |
| Pre-bonus payment period (t=2009 to 2011) | |  |  |  |
| Within effect: Star rating change within a contract | 255.8 (-4451.2, 4962.7) | 0.915 | 771.7 (-1915.7, 3459.1) | 0.573 |
| Post-bonus payment period (t=2012 to 2015) | |  |  |  |
| Within effect: Star rating change within a contract | 3487.7 (-125.5, 7101.0) | 0.058 | 11,292.6 (5940.1, 16,645.1) | <0.001 |
| Pre-post combined period (t=2009 to 2015) | |  |  |  |
| Within effect * Post^a^ | 1897.8 (-1059.3, 4854.8) | 0.208 | 3411.7 (480.3, 6343.2) | 0.023 |

CI, confidence interval; MAPD, Medicare Advantage Prescription Drug plan.

^a^ Post=1 if year was from 2012 to 2015 and Post=0 if year was from 2009 to 2011. Estimates were based on fixed effects models adjusted for contract-level repeated measures and controlling for the lagged (t-1 year) variables of total number of plans in the contract, proportion of plans in the contract that offered Part D coverage, proportion of plans in the contract that were Special Needs Plans (i.e., those that limit membership to people with specific diseases or characteristics, and tailor their benefits, provider choices, and drug formularies to best meet the specific needs of the groups they serve), and the proportion of plans in the contract that were employer group health plans in the prior (t-1) year, and year dummy variables. “Within effect * Post” is the interaction term between “Within effect” and “Post.”

Table B. Sensitivity Analysis on Sample Selection: Association of Star Rating and MAPD Contract Enrollment Before and After 2012 (Panel Data Hybrid Model; **Including** **Medicare Cost Contracts** in the Sample) ^a^

|  | Enrollment in concurrent year (t) | | Enrollment in subsequent year (t+1) | |
| --- | --- | --- | --- | --- |
|  | Estimate (95% CI) | P-value | Estimate (95% CI) | P-value |
| Pre-bonus payment period (t=2009 to 2011) | |  |  |  |
| Between effect: Star rating difference between contracts | 7858.8 (-335.2, 16,052.7) | 0.060 | 9280.1 (364.2, 18,195.9) | 0.041 |
| Within effect: Star rating change within a contract | 148.4 (-4734.0, 5030.9) | 0.952 | 692.5 (-2109.4, 3494.4) | 0.628 |
| Post-bonus payment period (t=2012 to 2015) | |  |  |  |
| Between effect: Star rating difference between contracts | 8688.8 (578.6, 16,798.9) | 0.036 | 11,347.3 (1553.8, 21,140.8) | 0.023 |
| Within effect: Star rating change within a contract | 3558.3 (-0.6, 7117.1) | 0.050 | 11,222.4 (5993.8, 16,450.9) | <0.001 |
| Pre-post combined period (t=2009 to 2015) | |  |  |  |
| Between effect: Star rating difference between contracts | 8577.1 (818.4, 16,335.8) | 0.030 | 10,423.6 (1520.3, 19,327.0) | 0.022 |
| Within effect * Post^a^ | 1562.0 (-4938.9, 8062.9) | 0.638 | 8989.4 (2017.7, 15,961.0) | 0.011 |

CI, confidence interval; MAPD, Medicare Advantage Prescription Drug plan.

^a^ Post=1 if year was from 2012 to 2015 and Post=0 if year was from 2009 to 2011. Estimates were based on hybrid models adjusted for contract-level repeated measures and controlling for contract types (health maintenance organization [HMO], point-of-service [POS], local provider organization [PPO], private fee-for-service [PFFS], regional PPO, or Medicare Cost), contract maturity (how many years the contract has been in existence, defined as the time elapsed between the year the contract became effective and the study year), and the lagged (t-1 year) variables of total number of plans in the contract, proportion of plans in the contract that offered Part D coverage, proportion of plans in the contract that were Special Needs Plans (i.e., those that limit membership to people with specific diseases or characteristics, and tailor their benefits, provider choices, and drug formularies to best meet the specific needs of the groups they serve), and the proportion of plans in the contract that were employer group health plans in the prior (t-1) year, and year dummy variables. “Within effect * Post” is the interaction term between “Within effect” and “Post.”

Table C. Sensitivity Analysis on Sample Selection: Association of Star Rating and MAPD Contract Enrollment Before and After 2012 (Panel Data Hybrid Model; **Excluding PFFS** From the Sample)^a^

|  | Enrollment in concurrent year (t) | | Enrollment in subsequent year (t+1) | |
| --- | --- | --- | --- | --- |
|  | Estimate (95% CI) | P-value | Estimate (95% CI) | P-value |
| Pre-bonus payment period (t=2009 to 2011) | |  |  |  |
| Between effect: Star rating difference between contracts | 9976.9 (1885.7, 18,068.1) | 0.016 | 11,197.6 (2294.0, 20,101.1) | 0.014 |
| Within effect: Star rating change within a contract | 473.1 (-2732.5, 3678.6) | 0.772 | 971.3 (-1793.7, 3736.3) | 0.491 |
| Post-bonus payment period (t=2012 to 2015) | |  |  |  |
| Between effect: Star rating difference between contracts | 9108.7 (869.1, 17,348.4) | 0.030 | 11,681.5 (1806.1, 21,556.8) | 0.020 |
| Within effect: Star rating change within a contract | 3836.1 (141.3, 7530.8) | 0.042 | 12,117.9 (6687.3, 17,548.5) | <0.001 |
| Model include 2009 to 2015 | |  |  |  |
| Between effect: Star rating difference between contracts | 9235.5 (1580.3, 16,890.7) | 0.018 | 10,984.4 (2082.3, 19,886.5) | 0.016 |
| Within effect * Post^a^ | 1366.4 (-5040.7, 7773.5) | 0.676 | 9410.9 (2300.8, 16,521.0) | 0.009 |

CI, confidence interval; MAPD, Medicare Advantage Prescription Drug plan.

^a^Post=1 if year was from 2012 to 2015 and Post=0 if year was from 2009 to 2011. Estimates were based on random effects models adjusted for contract-level repeated measures and controlling for contract types (health maintenance organization [HMO], point-of-service [POS], local provider organization [PPO], or regional PPO), contract maturity (how many years the contract has been in existence, defined as the time elapsed between the year the contract became effective and the study year), and the lagged (t-1 year) variables of total number of plans in the contract, proportion of plans in the contract that offered Part D coverage, proportion of plans in the contract that were Special Needs Plans (i.e., those that limit membership to people with specific diseases or characteristics, and tailor their benefits, provider choices, and drug formularies to best meet the specific needs of the groups they serve), and the proportion of plans in the contract that were employer group health plans in the prior (t-1) year, and year dummy variables. “Within effect * Post” is the interaction term between “Within effect” and “Post.”

Table D. Sensitivity Analysis on Sample Selection: Association of Star Rating and MAPD Contract Enrollment Before and After 2012 (Panel Data Hybrid Model; **Excluding Contracts with High Percentage of Special Needs Plans or Employer Group Health Plans** From the Sample)^a^

|  | Enrollment in concurrent year (t) | | Enrollment in subsequent year (t+1) | |
| --- | --- | --- | --- | --- |
|  | Estimate (95% CI) | P-value | Estimate (95% CI) | P-value |
| Pre-bonus payment period (t=2009 to 2011) | |  |  |  |
| Between effect: Star rating difference between contracts | 13,817.9 (-340.2, 27,975.9) | 0.056 | 17,320.4 (2068.4, 32,572.4) | 0.026 |
| Within effect: Star rating change within a contract | -1336.7 (-9757.5, 7084.1) | 0.756 | 465.0 (-3139.2, 4069.2) | 0.800 |
| Post-bonus payment period (t=2012 to 2015) | |  |  |  |
| Between effect: Star rating difference between contracts | 15,692.8 (2822.3, 28,563.4) | 0.017 | 21,419.3 (4929.0, 37,909.5) | 0.011 |
| Within effect: Star rating change within a contract | 6871.2 (-527.0, 14,269.3) | 0.069 | 18,291.7 (7547.3, 29,036.1) | 0.001 |
| Model include 2009 to 2015 | |  |  |  |
| Between effect: Star rating difference between contracts | 14,376.3 (2826.7, 25,925.9) | 0.015 | 16,719.6 (3422.4, 30,016.7) | 0.014 |
| Within effect * Post^b^ | 2655.9 (-9389.3, 14,701.1) | 0.666 | 14,400.3 (1327.8, 27,472.8) | 0.031 |

CI, confidence interval; MAPD, Medicare Advantage Prescription Drug plan.

^a^Sample excluded contracts with ≥50% plans being special needs plans or employer group health plans.

^b^Post=1 if year was from 2012 to 2015 and Post=0 if year was from 2009 to 2011. Estimates were based on random effects models adjusted for contract-level repeated measures and controlling for contract types (health maintenance organization [HMO]), point-of-service [POS], local provider organization [PPO], private fee-for-service [PFFS], or regional PPO), contract maturity (how many years the contract has been in existence, defined as the time elapsed between the year the contract became effective and the study year), and the lagged (t-1 year) variables of total number of plans in the contract, proportion of plans in the contract that offered Part D coverage, proportion of plans in the contract that were Special Needs Plans (i.e., those that limit membership to people with specific diseases or characteristics, and tailor their benefits, provider choices, and drug formularies to best meet the specific needs of the groups they serve), and the proportion of plans in the contract that were employer group health plans in the prior (t-1) year, and year dummy variables. “Within effect * Post” is the interaction term between “Within effect” and “Post.”

Table E. Sensitivity Analysis on Unit of Analysis: Association of Star Rating and MAPD Plan Enrollment Before and After 2012 (Panel Data Hybrid Model; **Plan-Year Level Analysis**)^a^

|  | **Enrollment^b^ in concurrent year (t)** | | **Enrollment^b^ in subsequent year (t+1)** | |
| --- | --- | --- | --- | --- |
|  | Estimate (95% CI) | P-value | Estimate (95% CI) | P-value |
| Without controlling for premium | |  |  |  |
| Pre-bonus payment period (t=2009 to 2011) | |  |  |  |
| Within effect: Star rating change within a contract | -54.0 (-541.8, 433.9) | 0.828 | -365.8 (-917.8, 186.1) | 0.194 |
| Post-bonus payment period (t=2012 to 2015) | |  |  |  |
| Within effect: Star rating change within a contract | 70.0 (-250.2, 390.2) | 0.668 | 461.4 (1.3, 921.6) | 0.049 |
| Pre-post combined period (t=2009 to 2015) | |  |  |  |
| Within effect * Post^c^ | 177.6 (-542.5, 897.8) | 0.629 | 711.9 (-116.8, 1540.6) | 0.092 |
| Controlling for premium^d^ | |  |  |  |
| Pre-bonus payment period (t=2009 to 2011) | |  |  |  |
| Within effect: Star rating change within a contract | -21.8 (-509.8, 466.1) | 0.930 | -328.0 (-879.6, 223.7) | 0.244 |
| Post-bonus payment period (t=2012 to 2015) | |  |  |  |
| Within effect: Star rating change within a contract | 22.1 (-293.1, 337.4) | 0.891 | 255.7 (-203.1, 714.6) | 0.275 |
| Pre-post combined period (t=2009 to 2015) | |  |  |  |
| Within effect * Post^c^ | 113.6 (-600.7, 827.8) | 0.755 | 547.0 (-274.0, 1368.0) | 0.192 |

CI, confidence interval; MAPD, Medicare Advantage Prescription Drug plan.

^a^Special needs plans or employer group health plans were excluded from the sample.

^b^Enrollment was set as missing in the original file if total enrollment was less than 11. We replaced this value with “5” in the analysis. The results were similar if missing values were replaced with “0” or “10.”

^c^Post=1 if year was from 2012 to 2015 and Post=0 if year was from 2009 to 2011. Estimates were based on hybrid models adjusted for plan-level repeated measures and controlling for plan types (health maintenance organization [HMO], point-of-service [POS], local provider organization [PPO], private fee-for-service [PFFS], or regional PPO), or premium amount [when included]), contract maturity (how many years the contract has been in existence, defined as the time elapsed between the year the contract became effective and the study year) and year dummy variables. “Within effect * Post” is the interaction term between “Within effect” and “Post.”

^d^Premium includes Part C premium plus Part D premium. Lower premium was associated with increased enrollment (data not shown).

Table F. Post-hoc Analysis: Association of Star Rating and MAPD Plan Premium Before and After 2012 (Panel Data Hybrid Model; **Plan-Year Level Analysis**)^a^

|  | **Premium^b^ in concurrent year (t)** | | **Premium^b^ in subsequent year (t+1)** | |
| --- | --- | --- | --- | --- |
|  | Estimate (95% CI) | P-value | Estimate (95% CI) | P-value |
| Pre-bonus payment period (t=2009 to 2011) | |  |  |  |
| Within effect: Star rating change within a contract | 1.6 (-0.4, 3.7) | 0.118 | 1.5 (-0.2, 3.1) | 0.079 |
| Post-bonus payment period (t=2012 to 2015) | |  |  |  |
| Within effect: Star rating change within a contract | -0.8 (-2.1, 0.4) | 0.183 | -4.8 (-6.5, -3.1) | <0.001 |
| Pre-post combined period (t=2009 to 2015) | |  |  |  |
| Within effect * Post^c^ | -0.9 (-3.3, 1.6) | 0.485 | -3.8 (-6.6, -1.1) | 0.006 |

CI, confidence interval; MAPD, Medicare Advantage Prescription Drug plan.

^a^Special needs plans or employer group health plans were excluded from the sample.

^b^Premium includes Part C premium plus Part D premium.

^c^Post=1 if year was from 2012 to 2015 and Post=0 if year was from 2009 to 2011. Estimates were based on hybrid models adjusted for plan-level repeated measures and controlling for plan types (health maintenance organization [HMO], point-of-service [POS], local provider organization [PPO], private fee-for-service [PFFS], or regional PPO), or premium amount [when included]), contract maturity (how many years the contract has been in existence, defined as the time elapsed between the year the contract became effective and the study year) and year dummy variables. “Within effect * Post” is the interaction term between “Within effect” and “Post.”

(A1)

(A2)

**Fig A. Stand-alone prescription drug plan (PDP) contracts, Star Ratings and Enrollment: 2009 to 2015**

(A1) Stand-alone prescription drug plan (PDP) contracts, Contract Mean Star Ratings and Distribution by Star Rating; (A2) Stand-alone prescription drug plan (PDP) contracts, Enrollment Distribution by Plan Rating

Table G. Analysis on External Comparison Group: Association of Star Rating and **Stand-Alone PDP Contract** Enrollment Before and After 2012 (Panel Data Hybrid Model)^a^

|  | Enrollment in concurrent year (t) | | Enrollment in subsequent year (t+1) | |
| --- | --- | --- | --- | --- |
|  | Estimate (95% CI) | P-value | Estimate (95% CI) | P-value |
| Pre-bonus payment period (t=2009 to 2011) | |  |  |  |
| Between effect: Star rating difference between contracts | 71,405.1 (-157,218.1, 300,028.3) | 0.540 | 40,117.4 (-266,568.0, 346,802.8) | 0.798 |
| Within effect: Star rating change within a contract | -22,772.9 (-152,453.1, 106,907.3) | 0.731 | -6,556.2 (-85,575.7, 72,463.2) | 0.871 |
| Post-bonus payment period (t=2012 to 2015) | |  |  |  |
| Between effect: Star rating difference between contracts | 166,168.0 (-44,373.9, 376,709.8) | 0.122 | 108,722.3 (-151,274.8, 368,719.4) | 0.412 |
| Within effect: Star rating change within a contract | -56,861.3 (-137,940.4, 24,217.9) | 0.169 | -30,794.5 (-89,128.2, 27,539.3) | 0.301 |
| Pre-post combined period (t=2009 to 2015) | |  |  |  |
| Between effect: Star rating difference between contracts | 96,604.9 (-157,073.7, 350,283.6) | 0.455 | 135,658.0 (-50,767.1, 322,083.1) | 0.154 |
| Within effect * Post^a^ | -11,193.5 (-203,500.7, 181,113.8) | 0.909 | 29,599.6 (-171,709.2, 230,908.4) | 0.773 |

CI, confidence interval; PDP, Prescription Drug Plan.

^a^Post=1 if year was from 2012 to 2015 and Post=0 if year was from 2009 to 2011. Estimates were based on hybrid models adjusted for contract-level repeated measures and controlling for contract maturity (how many years the contract has been in existence, defined as the time elapsed between the year the contract became effective and the study year), and the lagged (t-1 year) variables of total number of plans in the contract, proportion of plans in the contract that were Special Needs Plans (i.e., those that limit membership to people with specific diseases or characteristics, and tailor their benefits, provider choices, and drug formularies to best meet the specific needs of the groups they serve), and the proportion of plans in the contract that were employer group health plans in the prior (t-1) year, and year dummy variables. “Within effect * Post” is the interaction term between “Within effect” and “Post.”
